# AC Electroluminescent Processes in Pr^3+^-Activated (Ba_0.4_Ca_0.6_)TiO_3_ Diphase Polycrystals

**DOI:** 10.3390/ma10050565

**Published:** 2017-05-21

**Authors:** Nan Gao, Min Zhang, Jun-Cheng Zhang

**Affiliations:** 1 College of Physics, Qingdao University, Qingdao 266071, China; gaonan_qddx@163.com; 2 Laboratory for Regional Oceanography and Numerical Modeling, Qingdao National Laboratory for Marine Science and Technology, Qingdao 266237, China; zhangmin@fio.org.cn; 3 First Institute of Oceanography, State Oceanic Administration, Qingdao 266061, China

**Keywords:** powders, solid-state reaction, optical properties, functional applications

## Abstract

We investigated the properties of alternating current (AC)-driven electroluminescence from (Ba_0.4_Ca_0.6_)TiO_3_:Pr^3+^ diphase polycrystal-based device. The results of crystal phases and micrographs, and the symmetrical dual emissions in one AC cycle, indicate the spontaneous formation of a dielectric/phosphor/dielectric sandwich microstructure in (Ba_0.4_Ca_0.6_)TiO_3_:Pr^3+^. The electroluminescent device emits a red light of 617 nm, which is attributed to the ^1^D_2_-^3^H_4_ transition of Pr^3+^ in the phosphor phase. At a fixed AC frequency, the intensity of electroluminescence exhibits a steep enhancement when applying an increased driving electric field that is beyond a threshold. In a fixed driving electric field, the intensity of electroluminescence shows a rapid rise at low frequencies, but reaches saturation at high frequencies. Based on a double-injection model, we discussed systematically the electroluminescent processes in a whole cycle of AC electric field, which matched well with the experimental data. Our investigation is expected to expand our understanding of such a diphase electroluminescent device, thereby promoting their applications in lighting and displays.

## 1. Introduction

Since the electroluminescence was first discovered in 1936 in the ZnS-based device [1], a range of products based on AC powder electroluminescence have been developed and applied, including the typical application in flat-panel displays and solid-state lighting [2,3,4,5]. However, most of commercial electroluminescent devices are generally prepared by the complex technique and cumbersome steps to construct a multilayer stacking structure, such as the doubly insulated AC thin electroluminescent devices [6,7,8,9,10]. The dielectric layers are used to enhance the resistance of devices to a high electric field and to provide the initial injection electron into phosphor layers. Wang et al. reported the electroluminescent phenomenon in multifunctional (Ba_1−*x*_Ca*_x_*)TiO_3_:Pr^3+^ (0.25 ≤ *x* ≤ 0.90) diphase polycrystals that are composed of Ba_0.77_Ca_0.23_TiO_3_:Pr^3+^ dielectric grains and Ba_0.1_Ca_0.9_TiO_3_:Pr^3+^ phosphor grains [11]. Notably, their research indicated that a series of dielectric/phosphor/dielectric sandwich architectures was formed spontaneously on a micrometer scale during the material synthesis through a conventional solid-state reaction, which greatly simplifies the manufacturing processes of electroluminescent devices and raises the possibility of developing novel devices [12]. Zhang et al. subsequently ascribed the injection of initial electrons from dielectric grain to phosphor grain to the Schottky emission of the interface state in the high electric field [13]. However, to the best of our knowledge, there have not been related reports that elucidate the electroluminescent process of such a diphase system during a complete AC cycle, which hinders the promotion of application to some extent. In the present paper, we employed the composition of (Ba_0.4_Ca_0.6_)TiO_3_:Pr^3+^ with the optimal electroluminescence (see Reference [12]) as the model material to systematically investigate the detailed electroluminescent properties and to further reveal the processes of electroluminescence from diphase (Ba, Ca)TiO_3_:Pr^3+^. Our results are expected to deepen our understanding on such a diphase electroluminescent device and to promote the future utilization in the field of lighting and displays.

## 2. Results and Discussion

**Figure 1:**
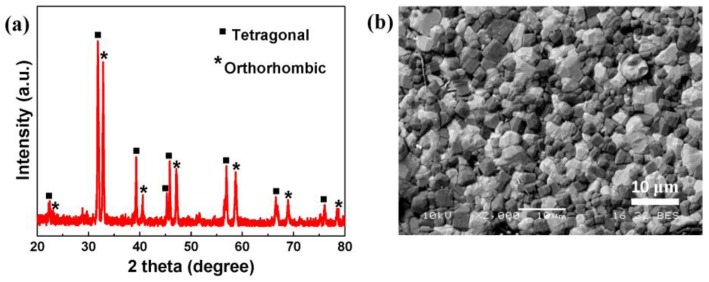
(**a**) XRD pattern of (Ba_0.4_Ca_0.6_)TiO_3_:Pr^3+^ powders; (**b**) backscattered-SEM picture of (Ba_0.4_Ca_0.6_)TiO_3_:Pr^3+^ pellet.

Figure 1a presents the powder X-ray diffractometer (XRD) pattern of (Ba_0.4_Ca_0.6_)TiO_3_:Pr^3+^, revealing a diphase coexistence of tetragonal Ba_0.77_Ca_0.23_TiO_3_:Pr^3+^ phase and orthorhombic Ba_0.1_Ca_0.9_TiO_3_:Pr^3+^ phase [11]. The backscattered-scanning electron microscopy (SEM) image as shown in Figure 1b exhibits obviously two types of polycrystal composites, attesting the analysis of XRD result. The dark Ba_0.1_Ca_0.9_TiO_3_:Pr^3+^ phosphor grains are tightly sandwiched by light Ba_0.77_Ca_0.23_TiO_3_:Pr^3+^ dielectric grains, and this fact supports the proposed dielectric/phosphor/dielectric sandwich microstructure for the (Ba_0.4_Ca_0.6_)TiO_3_:Pr^3+^ diphase polycrystals [12].

**Figure 2:**
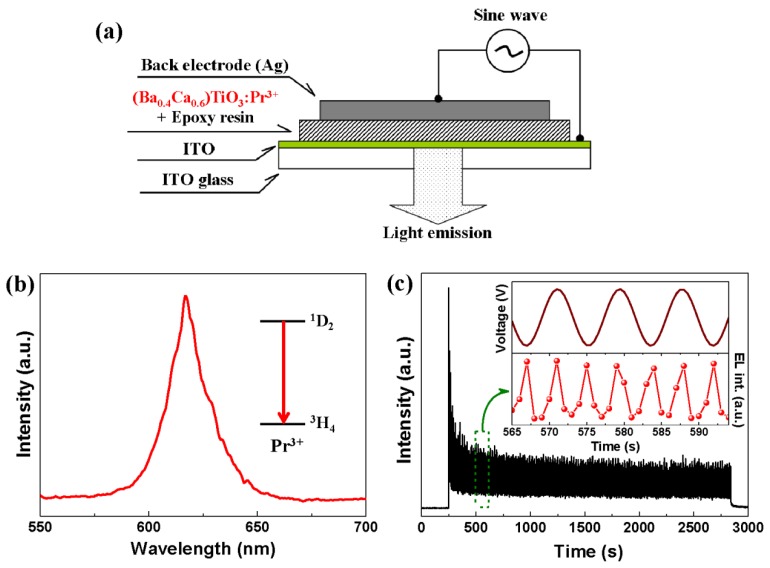
(**a**) Schematic structure of AC electroluminescent device based on (Ba_0.4_Ca_0.6_)TiO_3_:Pr^3+^; (**b**) electroluminescent spectra of (Ba_0.4_Ca_0.6_)TiO_3_:Pr^3+^-based device, showing the characteristic emission of Pr^3+^ of ^1^D_2_-^3^H_4_ transition; (**c**) electroluminescent behavior of (Ba_0.4_Ca_0.6_)TiO_3_:Pr^3+^-based device in an AC driving field (700 V, 60 Hz). The inset shows the enlarged, time-resolved electroluminescent response. ITO: indium tin oxide.

Figure 2a exhibits the schematic structure of the AC electroluminescent device composed of (Ba_0.4_Ca_0.6_)TiO_3_:Pr^3+^ powders and epoxy resin. When applying an AC electric field on the device via the top Ag electrode and the bottom indium tin oxide (ITO) electrode, the electroluminescence could be observed and measured simultaneously from the bottom optical ITO glass. Figure 2b shows the electroluminescent spectrum of the (Ba_0.4_Ca_0.6_)TiO_3_:Pr^3+^-based device. Only one spectral peak located at 617 nm was observed, which is attributed to the ^1^D_2_-^3^H_4_ transition of Pr^3+^ in the phosphor phase [14]. Figure 2c presents the transient response of electroluminescent signals. It demonstrates an attenuation when applying the AC electric field initially and is followed by a relatively stable flatform under the sustained excitation of electric field. The enlarged, time-resolved emission response of the flatform stage is shown in the inset of Figure 2c, which provides the detailed characteristics of electroluminescent device. In each cycle of the AC electric field, the electroluminescent signals consist of two emission peaks that correspond to the positive and negative peaks of AC voltage, respectively. The intensity ratio of two arbitrary adjacent emission peaks is nearly 1. This result is consistent with the luminescent feature of thin film electroluminescent devices (TFELDs) with typical symmetrical structure [9,15], supporting again our proposal of a dielectric/phosphor/dielectric sandwich microstructure.

**Figure 3:**
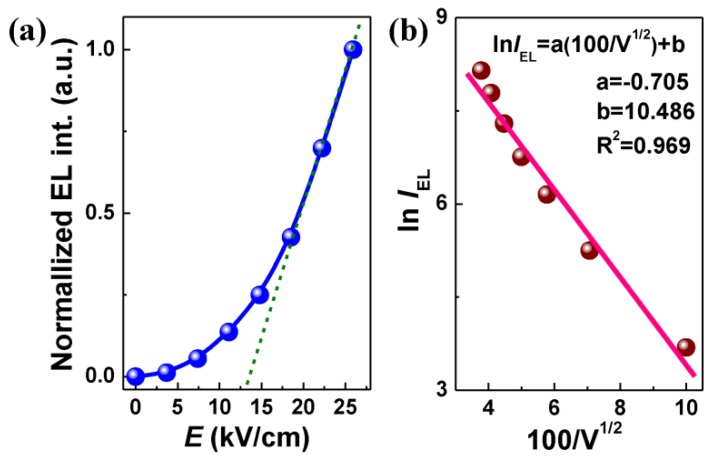
(**a**) Electric field (*E*) dependence of electroluminescent intensity for (Ba_0.4_Ca_0.6_)TiO_3_:Pr^3+^-based device; (**b**) relation of the electroluminescent intensity (ln*I*_EL_) and the applied voltage (100/*V*^1/2^).

Figure 3a illustrates the dependence of electroluminescent intensity on an increasing electric field at a fixed frequency of 60 Hz. It shows a relatively slow increase at a lower electric field, but a steep enhancement beyond a threshold of ~13 kV/cm. This trend is also similar to the case of TFEDLs employing lanthanide-doped phosphors as the luminescent layers [9,15,16]. Figure 3b plots the relation of electroluminescent intensity (*I*_EL_) and applied voltage (100/*V*^1/2^). The experimental data basically match the well-known empirical equation of *L* = *L*_0_exp(m*V*^−1/2^), where *L* is the luminance, *V* is the applied voltage, and *L*_0_ and m are constants that are determined by the phosphor material, device structure, and test condition [17].

**Figure 4:**
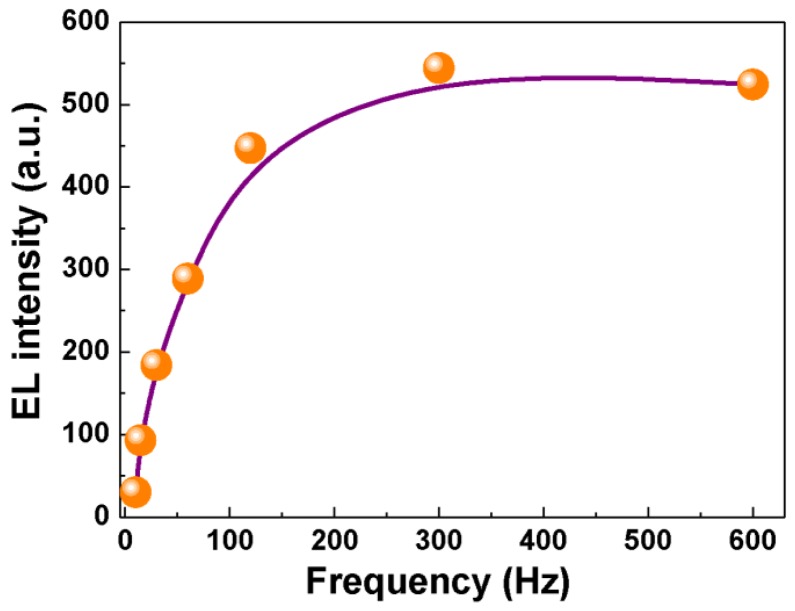
Dependence of electroluminescent intensity on the frequency of the AC electric field.

We also investigated the dependence of electroluminescence on the frequency (10–600 Hz) at a fixed AC driving voltage (700 V), as depicted in Figure 4. The electroluminescent intensity presents a rapid rise at relatively low frequencies (0–120 Hz) and demonstrates a saturated trend with further enlargement in frequency (120–600 Hz).

**Figure 5:**
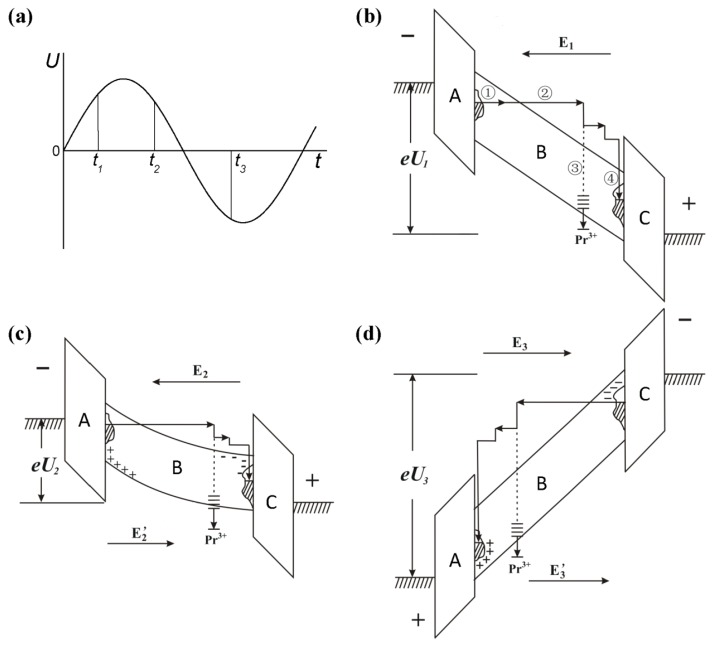
Schematic illustration of the proposed AC electroluminescent processes in (Ba_0.4_Ca_0.6_)TiO_3_:Pr^3+^ diphase polycrystals with a sandwich structure. A and C: Ba_0.77_Ca_0.23_TiO_3_:Pr^3+^ dielectric grains; B: Ba_0.1_Ca_0.9_TiO_3_:Pr^3+^ phosphor grain. **(a)** Three representative moments (*t*_1_, *t*_2_, *t*_3_) in a cycle of AC electric field, **(b)**–**(d)** electroluminescent processes corresponding to *t*_1_, *t*_2_, *t*_3_ moments, respectively. ①: Schottky emission; ②: electron accelerating; ③: impact excitation; ④: interface trapping.

Based on the above-mentioned investigation on the characteristics of electroluminescence and our previous results [11,12,13,14], the AC electroluminescent mechanism in (Ba_0.4_Ca_0.6_)TiO_3_:Pr^3+^ diphase polycrystals could be explained by a double-injection model [18]. Figure 5 schematically illustrates the electroluminescent processes in a complete cycle of AC electric field, taking three representative moments as examples (Figure 5a).

First, the electroluminescence at the moment of *t*_1_ might be divided into the following four steps (Figure 5b). When the increased voltage exceeds the threshold of electroluminescence (Figure 3a), the initial electrons trapped in the interface state of dielectric layer A and phosphor layer B are emitted into a phosphor grain by the Schottky emission [13] (Step 1). Under the external electric field of E_1_, these electrons are unceasingly accelerated to become hot electrons with enough kinetic energy (Step 2). The hot electrons subsequently impact and excite the luminescent center Pr^3+^, which is followed by a light emission (electroluminescence) induced by the de-excitation of Pr^3+^. The hot electrons could also result in the impact ionization of electrons in the valence band of the phosphor layer to form space charges (electrons and holes) (Step 3). The rest of the hot electrons and the new space electrons might reach the interface of B and C and then be captured by the interface state, while the space holes could be trapped at the interface state of A and B (Step 4).

Second, at the moment of *t*_2_ when the applied voltage exceeds the peak value and is further decreased (Figure 3a), a reversed electric field (E_2_’) is generated by the accumulated space charges (Figure 5c). It further weakens the externally applied electric field (E_2_), leading to a prompt decrease in electroluminescence. Accordingly, an emission peak of electroluminescence could be observed during the positive half cycle of the AC electric field (Figure 2c).

Third, at the moment of *t*_3_ when the applied voltage is reversed (Figure 3a), the additional electric field (E_3_’) created by the space charges shows the same direction as does the external electric field (E_3_) (Figure 5d). The dual electric field is applied on the phosphor layer, resulting in a similar process with Figure 5b. Therefore, another emission peak would also be observed during the negative half cycle of the AC electric field (Figure 2c).

Furthermore, this model can also explain the saturation of electroluminescence under the high frequency of electric fields. The reason is that the direction of the applied electric field is reversed too quickly for the electrons to complete the process involving Steps 1–4.

## 3. Materials and Methods

Pr^3+^-activated (Ba_0.4_Ca_0.6_)TiO_3_ diphase polycrystals were synthesized through a solid-state reaction according to a stoichiometric composition of (Ba_0.4_Ca_0.6_)_0.998_Pr_0.002_TiO_3_ (denoted as (Ba_0.4_Ca_0.6_)TiO_3_:Pr^3+^ for simplicity). Raw materials of BaCO_3_, CaCO_3_, Pr_6_O_11_, and TiO_2_ (≥ 99.9%) were thoroughly ground and calcined at 900 °C for 4 h in air, reground, and then partially pelletized. Subsequently, the pellets and powders were sintered at 1400 °C for 4 h in air. The sintered pellets were collected for examination by SEM, while the powder samples were ground and screened through a 20 μm sieve for the preparation of the electroluminescent device.

The crystallization behavior was examined by an XRD (D8 Advance, Bruker AXS, Karlsruhe, Germany) and a field-emission SEM (JSM-5510, JEOL, Tokyo, Japan). The electroluminescent device was prepared by the following procedure. The mixture of screened powders and optical epoxy resin (weight ratio of 3:1) was printed on the surface of an ITO glass to form an electroluminescent layer with an area of 10 mm × 10 mm and a thickness of 0.3 mm. Silver conducting paste was brushed on the surface of an electroluminescent layer. An AC electric power supply (HP3300A, Hewlett-Packard, Palo Alto, California) and an amplifier (TREK677B, TREK, Wisconsin, USA) were used to supply the driving electric field. The electroluminescent signals were measured by a photon-counting system that consists of a photomultiplier tube (R649, Hamamatsu Photonics, Hamamatsu city, Japan) and a photocounter (C3866, Hamamatsu Photonics, Hamamatsu city, Japan) controlled by a computer. The electroluminescent spectrum was recorded by a photon multi-channel analyzer system (PMA-100, Hamamatsu Photonics). All measurements were performed at room temperature.

## 4. Conclusions

The AC electroluminescent properties of a (Ba_0.4_Ca_0.6_)TiO_3_:Pr^3+^-based device have been investigated. The results of XRD and backscattered-SEM reveal a diphase coexistence of a Ba_0.77_Ca_0.23_TiO_3_:Pr^3+^ dielectric phase and a Ba_0.1_Ca_0.9_TiO_3_:Pr^3+^ phosphor phase in (Ba_0.4_Ca_0.6_)TiO_3_:Pr^3+^ polycrystals. The symmetrical dual emissions in one cycle of an AC-driven electric field support the proposed dielectric/phosphor/dielectric sandwich microstructure. The red emission located at 617 nm was attributed to the ^1^D_2_-^3^H_4_ transition of Pr^3+^ in the phosphor phase. The intensity of electroluminescence exhibits a steep enhancement as increasing the driving electric field beyond a threshold of ~13 kV/cm. Furthermore, the relationship of electroluminescence and applied voltage matches the well-known empirical equation of *L* = *L*_0_exp(m*V*^−1/2^). On the other hand, the electroluminescent intensity shows a rapid rise at low frequencies (0–120 Hz), but reaches saturation at high frequencies (120–600 Hz). Finally, the electroluminescent processes in an AC cycle have been systematically discussed according to a double-injection model. After the systematical investigation and comprehension, we expect to promote the further application of such a device in lighting and displays.
